# Linking population‐level and microevolutionary processes to understand speciation dynamics at the macroevolutionary scale

**DOI:** 10.1002/ece3.7511

**Published:** 2021-05-01

**Authors:** Laura Rodrigues Vieira de Alencar, Tiago Bosisio Quental

**Affiliations:** ^1^ Departamento de Ecologia Instituto de Biociências Universidade de São Paulo São Paulo Brazil

**Keywords:** dispersion ability, geographic opportunities, macroevolution, microevolution, reproductive isolation, speciation

## Abstract

Although speciation dynamics have been described for several taxonomic groups in distinct geographic regions, most macroevolutionary studies still lack a detailed mechanistic view on how or why speciation rates change. To help partially fill this gap, we suggest that the interaction between the time taken by a species to geographically expand and the time populations take to evolve reproductive isolation should be considered when we are trying to understand macroevolutionary patterns. We introduce a simple conceptual index to guide our discussion on how demographic and microevolutionary processes might produce speciation dynamics at macroevolutionary scales. Our framework is developed under different scenarios: when speciation is mediated by geographical or resource‐partitioning opportunities, and when diversity is limited or not. We also discuss how organismal intrinsic properties and different overall geographical settings can influence the tempo and mode of speciation. We argue that specific conditions observed at the microscale might produce a pulse in speciation rates even without a pulse in either climate or physical barriers. We also propose a hypothesis to reconcile the apparent inconsistency between speciation measured at the microscale and macroscale, and emphasize that diversification rates are better seen as an emergent property. We hope to bring the reader's attention to interesting mechanisms to be further studied, to motivate the development of new theoretical models that connect microevolution and macroevolution, and to inspire new empirical and methodological approaches to more adequately investigate speciation dynamics either using neontological or paleontological data.

## Graphical abstract

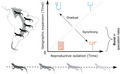

## 1 INTRODUCTION: THE MECHANISTIC GAP IN MACROEVOLUTION

When populations experience different selective pressures or randomly accumulate genetic differences, they might become so distinct from each other that they may no longer produce viable offspring or even no longer reproduce (Coyne & Orr, 2004; Mayr, 1942). This scenario would result in the formation of new species (biological species concept) and illustrates the process of speciation. If we scale up this process to the macroevolutionary scale, the formation of new species should leave detectable signals in the diversity trajectories of a clade, and the speciation dynamics can be documented and studied. Macroevolutionary biologists capture these signals by measuring how speciation rates (number of lineages generated per lineage per million years) vary both along time and among clades. Hence, to understand how biodiversity originates and is maintained, one needs to describe how speciation varies and what are the mechanisms underlying such dynamics.

It has been known for quite a while that differences in speciation rates among lineages might be related to differences in dispersal abilities (e.g., Hansen, 1983; Jablonski, 1986) and ecological specialization (e.g., Vrba, 1987). Since then speciation dynamics have been described for a range of taxonomic groups or geographic regions (e.g., Cantalapiedra et al., 2017; Lovette & Bermingham, 1999; Morlon et al., 2012; Pires et al., 2015), and our knowledge on the underlying mechanisms of species radiations at macroevolutionary scales have strongly relied on the use of a series of statistical methods using both fossil and phylogenetic data. These modern statistical methods are frequently applied to infer potential associations between rates of speciation and traits (e.g., reproduction mode, Lynch, 2009; body size and temperature, Silvestro et al., 2015; Diet, Burin et al., 2016), between speciation rates and a time series describing a potential factor (e.g., temperature, Condamine et al., 2018; Pires et al., 2017), or to speculating what factors would underlie speciation rate variation through time (e.g., Morlon et al., 2010) or across the tree of life (e.g., Cantalapiedra et al., 2017). Moreover, the underlying mechanisms of such changes in speciation rates have been mostly discussed in terms of ecological opportunities, competitive interactions, and resource‐partitioning (e.g., Schluter, 2000; Burbrink & Pyron, 2009; Jønsson et al., 2012; Pires et al., 2015; García‐Navas et al., 2018 but see Fritz et al., 2011), discussions probably inspired by the iconic studies on adaptive radiations (e.g., Darwin's Finches, Grant & Grant, 2008; Caribbean Anoles, Losos, 2009; Cichlids, Seehausen, 2015).

Although the field of microevolution has greatly advanced in terms of our understanding of how reproductive isolation emerges and how new species are generated, those do not typically investigate how rates of speciation change through time. On the other hand, even though speciation dynamics is an important focus of many empirical macroevolutionary studies, those still lack a detailed population mechanistic view of how or why speciation rates change (but see Martin & Richards, 2019). This limitation is related to our general difficulty to link microevolutionary processes to macroevolutionary patterns (Erwin, 2000; Harvey et al., 2019; Jablonski, 2000; Pennell et al., 2014; Uyeda et al., 2011), an area that has received increasing interest in the past few years (e.g., Dynesius & Jansson, 2013; Harvey et al., 2017, 2019; Martin & Richards, 2019; Rabosky & Matute, 2013; Singhal et al., 2018; Uyeda et al., 2011). Despite this increasing interest and the recent theoretical effort on the matter (e.g., Aguilée et al., 2018; Costa et al., 2019), we still know little on how (and if) repeated rounds of microevolutionary events would translate into the patterns detected at the macroevolutionary scale.

To help partially fill these gaps, we discuss how the time taken by a species to geographically expand and the time its populations take to evolve reproductive isolation interact to produce gradual or (des)accelerated speciation dynamics observed at the macroevolutionary scale. Our discussion incorporates the potential role of environmental heterogeneity and resource partitioning in driving these diversification patterns. We also discuss how the intrinsic properties of the organisms and geographical settings might affect those population‐level and microevolutionary processes that ultimately change the macroevolutionary patterns. Our intention here is to highlight some interesting ideas and mechanisms that could help us to better link microevolutionary mechanisms to macroevolutionary patterns and not to encompass all possible mechanisms potentially involved in biodiversity evolution. By doing this, we hope to inspire the development of new mathematical models and foster new ways to empirically study biological systems.

## 2 A HEURISTIC INDEX TO LINK POPULATION‐LEVEL AND MICROEVOLUTIONARY PROCESSES TO PREDICT MACROEVOLUTIONARY PATTERNS

We first present a simple heuristic index (Equations 1 and 2) to guide our discussion on how the timing between population‐level mechanisms and the evolution of reproductive isolation interact at short‐time scales and translate into speciation dynamics at deeper time scales. We emphasize this is not a formal mathematical model, but a simple index that might eventually inspire a dynamic model. We define this index (here called Φ) as the ratio between what we consider to be important population and microevolutionary temporal quantities. Our index focuses on the interaction of elements such as the total habitable area, how populations move across space and use resources, and how long it takes for reproductive isolation to evolve among these populations.

(1) $$\Phi =\frac{t_{\exp}}{\text{TTBS}}$$

(2) $$\Phi =\frac{\frac{\text{Area}}{\text{Expansion rate}}}{\frac{N_{\text{diff}}}{\alpha +\beta }}$$

**FIGURE 1:**
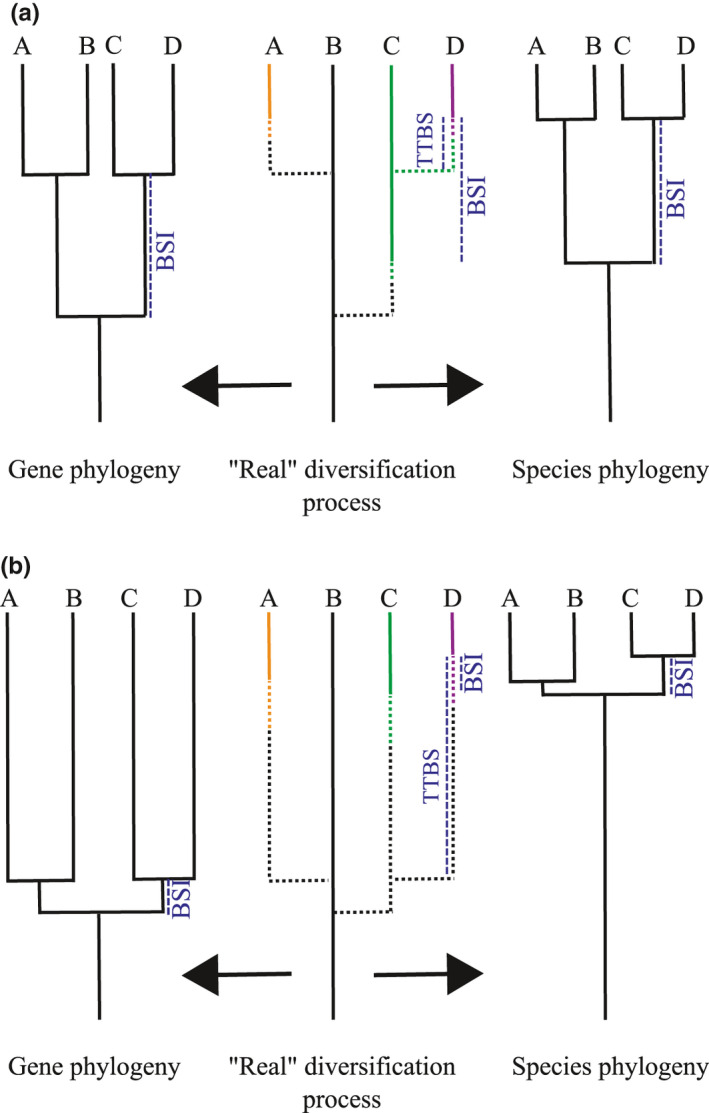
The biological speciation interval (BSI) and the transition time for biological speciation (TTBS) measured in examples of gradual (a) and accelerated (b) speciation dynamics (therefore, dynamics with relatively long vs. short BSI, respectively). In the middle, the “real” diversification process represents the real tempo of population and species splitting; on the left (Gene phylogeny), the splitting events capture the points in time where populations start to differentiate; on the right (Species phylogeny), the splitting events represent points in time where populations become true species according to the biological species concept. Dotted branch lengths represent the time since populations started to differentiate, and continuous branch lengths represent the time since reproductive isolation emerged. (a) Gradual diversification process: population splitting and the emergence of species occur roughly constant over time and TTBS is shorter than BSI. (b) An example of an accelerated diversification process where several population splits occur early in the clade's history but, in this specific case, species take a long time to become reproductively isolated (our “late burst” scenario, see Figure 2 and the text for details). Here TTBS is longer than BSI. Gene and species phylogenies would show, respectively, an early and late burst pattern of diversification at the macroevolutionary scale. For the sake of simplicity, we have assumed TTBS to be constant in absolute time and among lineages. Our arguments rest on the relative difference between TTBS and BSI, and hence would allow differences in TTBS either in time or among branches if this variation does not qualitatively change the relationship between TTBS and BSI (e.g., TTBS > BSI to TTBS < BSI). See also Coyne and Orr (2004) and Sukumaran and Knowles (2017)

The numerator of the index encapsulates different population‐level processes, and at its simplest form represents the time it takes for populations of a given species to attain the maximum geographical distribution available for the species (*t*_exp_ in Equation 1). The denominator encapsulates different microevolutionary processes, and at its simplest form represents the *transition time for biological speciation* (TTBS in Equation 1, see also the Glossary in Box 1, Coyne & Orr, 2004). It is important to note that TTBS is different from the *biological speciation interval* (BSI), another important temporal quantity (Coyne & Orr, 2004; see Box 1 and Figure 1).

**Glossary**

*Transition time for biological speciation (TTBS)*. Describes the time taken for reproductive isolation to be completed once a population starts to differentiate (see Coyne & Orr, 2004). This is equivalent to “speciation duration” or “population conversion” proposed by Dynesius and Jansson (2013) and Li et al. (2018), respectively. The TTBS concept implicitly incorporates the idea of protracted speciation (Etienne et al., 2014; Etienne & Rosindell, 2012; Rosindell et al., 2010).

*Biological speciation interval (BSI)*. Describes the waiting times between two successive speciation events during the radiation of a lineage (Coyne & Orr, 2004).

*Resource‐partitioning opportunities*. Plenty of different resources (e.g., hosts, food) conducive to sympatric speciation driven by competition that result in adaptive differentiation as a consequence of resource utilization (e.g., Martin & Feinstein, 2014; see also Martin & Richards, 2019). Similar to “ecological opportunities” defined by Schluter (2000), although here we explicitly imply coexistence. We give emphasis on competition as a driver of species divergence, although other biotic interactions such as predation (Endara et al., 2015), sexual selection (Kraaijeveld et al., 2011), as well as the interactions between different biotic mechanisms (Wagner et al., 2012), can also drive sympatric differentiation between populations.

*Geographic opportunities*. Plenty of conditions conducive to allopatric or parapatric speciation (Moen & Morlon, 2014; Rosenzweig, 1995). Different from resource‐partitioning opportunities, range expansion necessarily needs to occur for a species to access geographic opportunities. Geographic opportunities can be mediated by either environmental heterogeneity or by physical barriers. A scenario where speciation is driven by geographic opportunities mediated by environmental heterogeneity involves the range expansion of a species and the adaptation of its populations to spatially distributed adaptive peaks (e.g., Gavrilets & Vose, 2005; Wang & Bradburd, 2014). Those adaptive peaks could either reflect variability in resources, such as prey types, but also in habitat and climatic conditions (e.g., Walter et al., 2020). Here, speciation occurs in the presence of gene flow (parapatric speciation, Coyne & Orr, 2004; Rundell & Price, 2009). A scenario where speciation is driven by geographic opportunities mediated by physical barriers (e.g., rivers, mountains, Lagomarsino et al., 2016; O'Connell et al., 2017) involves the range expansion of a species and population differentiation driven by the interruption of gene flow (allopatric speciation, Coyne & Orr, 2004; Rundell & Price, 2009), potentially coupled with adaptations to the different isolated environments (e.g., Gray et al., 2019). Drift can also play a role when populations are differentiating across geographic opportunities, in particular when geographical opportunities are mediated by physical barriers. However, drift alone is unlikely to rapidly drive speciation, particularly when there is considerable gene flow (see Marie Curie Speciation Network, 2012).

*Incipient species*. Populations that are about to become “true” species. The same as “within‐species lineage” proposed by Dynesius and Jansson (2013).

*Ephemeral speciation*. Speciation model where species are rapidly produced but where most species do not persist over macroevolutionary timescales due to extinction or resorption (Dynesius & Jansson, 2013; Rosenblum et al., 2012).

*Protracted speciation*. Speciation is a gradual process in contrast to an instantaneous one. Under this model, incipient species are formed and could give rise to “true” species (those that persist). The process can hence be modeled with a “speciation‐initiation rate”, which could be different for incipient and “true” species, a “speciation‐completion” rate, which describes the rate at which incipient species turn into “true” species, and two rates of extinction, one for incipient species and another for “true” species (see Etienne et al., 2014; Etienne & Rosindell, 2012; Rosindell et al., 2010).

*Carrying capacity*. A cap on the number of species that is assumed under a specific scenario of equilibrium diversity where speciation and extinction rates are regulated by diversity‐dependent processes (Rabosky, 2013). For simplicity, we here view carrying capacity as a hard limit to richness but for a more complex view regarding equilibrium dynamics see Cornell (2013), Rabosky (2013), and Marshall & Quental (2016).

The numerator encapsulates how populations of a given species move across space, and it can be further decomposed into the total available area and the species' ability to expand geographically. Given the finite aspect of potential habitats available for a given species, there should be a “total habitable area” (“Area” in Equation 2; units being for instance square kilometers) (Herrera‐Alsina et al., 2018; Morrone, 2008). For simplicity, one could consider the total physical area (or the total area expected to be habitable given the organism's niche properties) as a proxy for this quantity. From the point of view of the birth (and death) of a new species, its initial (and final) geographical distribution is typically restricted, at least with respect to its maximum distribution typically attained at some time during its lifetime (Foote, 2007; Liow & Stenseth, 2007; Žliobaitė et al., 2017). Hence, the arrival of a species into a new area (e.g., Kennedy et al., 2018; Porto et al., 2021), the extinction of competitors (e.g., Silvestro et al., 2015), or the emergence of a key innovation (e.g., Matschiner et al., 2011), for example, might result in the dispersion of individuals into unoccupied areas. Given enough time, most of the available area might be occupied. How fast this might happen is described by the term “Expansion rate” (units being, for example, square kilometers per year). Hence in our formulation, the velocity of this range expansion is a crucial aspect to be studied. The interplay between the steepness of the environmental heterogeneity, the evolution of genetic variance, and genetic drift in the original population (Polechová, 2018; Polechová & Barton, 2015), together with the presence of competitors/predators in the area (Gavrilets & Vose, 2005; Herrera‐Alsina et al., 2018; Phillimore & Price, 2009), might determine whether the original population will successfully expand its spatial distribution. Additionally, species' dispersal ability is likely to affect such rates (see further discussion below). All those aspects can, and should, be further studied (see García‐Ramos & Rodríguez, 2002; Polechová, 2018; Polechová & Barton, 2015) but here we simplify those into a single rate (“Expansion rate” in our index; see Equation 2) to emphasize the demographic aspect that we think is relevant. Hence, the ratio of those two terms (“Area” and “Expansion rate”) results in the amount of time to reach the habitable area, “*t*_exp_,” our numerator in its simplest form. Given that speciation occurs in space (either sympatric or not), this term captures the effect that the geographical area might have on the likelihood of speciation, and, as we will see later, how it affects the pace of speciation.

The denominator represents the time it takes for reproductive isolation to be completed, and it can also be further decomposed. The term *N*_diff_ (units being the number of fixed differences, Equation 2) represents the number of differences (e.g., number of incompatible genetic loci; alleles adapted to different environments) that need to be fixed between populations (or metapopulations, or demes in the case of sympatric speciation) for a new species to be formed (Coyne & Orr, 2004; Gavrilets & Losos, 2009; Nosil, 2012). Hence, everything else being equal, the smaller the number of differences needed for reproductive isolation to occur, the faster new species will be formed (Gavrilets & Losos, 2009; Gavrilets & Vose, 2005). The term “(*α* + *β*)” represents the rate under which these differences accumulate (units being, for example, the number of fixed differences per year, Equation 2). To keep our framework simple, we have explicitly categorized this rate to reflect two general mechanisms: the first is the “*α*” term which relates to differences that emerge through resource‐partitioning opportunities (Box 1). In this case, differences between populations emerge as a product of divergent selection related to how individuals locally compete for resources and species formation would take place via sympatric speciation (e.g., Martin & Feinstein, 2014; see also Box 1). On the other hand, the “*β*” term relates to differences that emerge via geographic opportunities (Box 1). When a species (or group of species) starts to radiate, it typically increases its geographical range, reaching several geographical opportunities. Thus, range expansion necessarily needs to occur for the species to access these opportunities and for populations to differentiate across space (e.g., Gavrilets & Vose, 2005; Wang & Bradburd, 2014). Here, species formation would take place via parapatric and/or allopatric speciation, and both selection and drift can play a role (e.g., Marie Curie Speciation Network, 2012; Walter et al., 2020; O'Connell et al., 2017; Gray et al., 2019; see also Box 1). Thus, the ratio *N*_diff_/(*α* + *β*) determines the time it takes for the speciation process to be completed corresponding to the *transition time for biological speciation* (TTBS), our denominator in its simplest form.

Given our interest in speciation dynamics, we do not explicitly include extinction in our index, but there is no doubt that extinction is an extremely relevant process both at population as well as at species levels. Extinction dynamics at the population‐level might be particularly relevant when considering ephemeral speciation (Box 1; Cutter & Gray, 2016; Dynesius & Jansson, 2013; Rosenblum et al., 2012; Stanley, 1978). As pointed out by Rabosky (2013), the extinction of ephemeral species might suggest that speciation rates measured at macroevolutionary scales could be strongly determined by factors other than reproductive isolation, such as the persistence of those incipient ephemeral species (see also Dynesius & Jansson, 2013). Extinction dynamics at the species level is a crucial aspect of diversification dynamics although hard to be estimated when using molecular phylogenies (Louca & Pennell, 2020; Quental & Marshall, 2010; Rabosky, 2010). Therefore, we emphasize that our intention is not to directly predict what a molecular phylogeny will look like (e.g., Costa et al., 2019), but rather to discuss the “true” underlying diversification mechanisms and their macroevolutionary signals. That said, future work including extinction effects both at population and species levels is warranted (see Dynesius & Jansson, 2013; Etienne & Rosindell, 2012; Quental & Marshall, 2010; Rabosky, 2010, 2013; Rosindell et al., 2010).

## 3 THE INTERACTION BETWEEN POPULATION‐LEVEL AND MICROEVOLUTIONARY PROCESSES CAN CHANGE THE TEMPO AND MODE OF SPECIATION DYNAMICS

**FIGURE 2:**
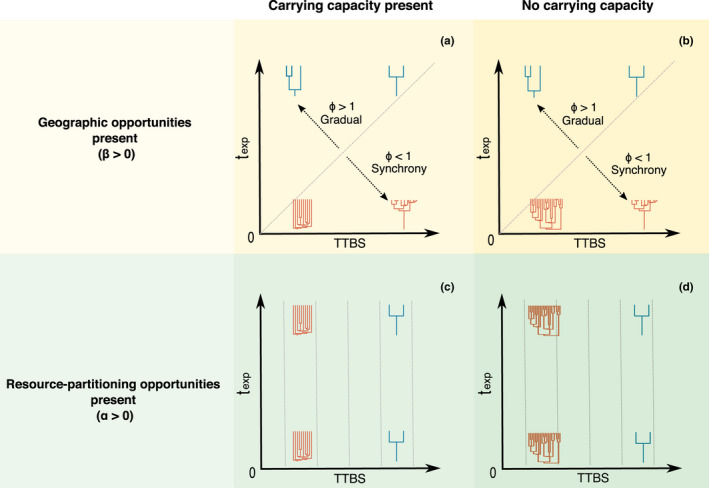
Different combinations between the time species take to geographically expand (*t*_exp_) and the time its populations take to evolve reproductive isolation (TTBS) (see Equations 1 and 2) might result in distinct speciation patterns observed at the macroevolutionary scale. (a) Speciation dynamics that are expected to emerge in a hypothetical scenario where geographic but not resource‐partitioning opportunities are present, under a species carrying capacity scenario. (b) The same as (a) but without carrying capacity. (c) Speciation dynamics that are expected to emerge in a hypothetical scenario where resource partitioning but not geographic opportunities are present, under a species carrying capacity scenario. (d) The same as (c) but without carrying capacity. All phylogenies were drawn considering that the same amount of time has elapsed from the time of origin. Each phylogeny is roughly placed in the graph according to the values of *t*_exp_ and TTBS that would generate it. Extinction rates are assumed to be zero (*μ* = 0). Phylogenies here represent “species phylogenies” where the branching times represent the time since species became fully isolated (BSI, Coyne & Orr, 2004)

The macroevolutionary patterns emerging from the distinct possible combinations between the time a species takes to geographically expand and the time its populations take to evolve reproductive isolation are the focus of this section and are illustrated in Figure 2 by the different shapes of hypothetical species phylogenies. To help the flow of our argument on how different processes can interact to produce empirical patterns, we discuss extreme scenarios even if those represent simplistic views of reality. As we will show, focusing on simple opposite scenarios can bring insights into the range of mechanisms possibly underlying increases in speciation rates when exploring the unfold of radiations. Therefore, we develop our framework separately for when speciation is mediated by geographical or resource‐partitioning opportunities and when species carrying capacity is present or not (Box 1).

Geographic and resource‐partitioning opportunities comprise together important aspects underlying radiations (e.g., Aguilée et al., 2018; Costa et al., 2019; Herrera‐Alsina et al., 2018; Kennedy et al., 2018; Stroud & Losos, 2016). As mentioned above, geographic opportunities are tied to the importance of the geographical area and range expansion in promoting speciation, and are probably underlying several radiations, including continental ones, from dinosaurs (O'Donovan et al., 2018) to plants (Walter et al., 2020). On the other hand, resource‐partitioning opportunities are frequently associated with sympatric speciation also contributing to the unfolding of radiations, especially those that are restricted in area, such as lake‐associated fishes (e.g., Kautt et al., 2012; Martin & Feinstein, 2014). The presence or absence of a carrying capacity regulating species diversity comprises a long debate in the macroevolutionary literature (see Box 1). On one hand, there are several examples of radiations showing diversity‐dependent diversification (Alroy, 1996, 2008; Rabosky, 2013; Rabosky & Hulbert, 2015; Sepkoski, 1981), an expected outcome of the presence of carrying capacity (although not an unequivocal evidence for the existence of a hard limit on diversity, Cornell, 2013; Harmon & Harrison, 2015; Marshall & Quental, 2016). On the other hand, some authors suggest that diversity trajectories might be better described by an unlimited increase in the number of species (Benton & Emerson, 2007; Harmon & Harrison, 2015; Stanley, 2007, 2008), or that the signal of a diversification slowdown might in fact be generated by statistical artifacts, taxon sampling, or given the prolonged nature of speciation (at least when analyzing molecular phylogenies, see Harmon & Harrison, 2015). Therefore, different macroevolutionary patterns can be expected if lower‐level processes interact in a world where diversity is bounded or unbounded. In Figure 2, we show the macroevolutionary patterns that might emerge when the interaction between population‐level and microevolutionary processes takes place in each of these different scenarios.

### 3.1 The tempo and mode of speciation dynamics driven by geographical opportunities

Both physical barriers and environmental heterogeneity could generate geographical opportunities (see Box 1) and, at least in theory, generate a pulse in speciation (see Moen & Morlon, 2014; Rangel et al., 2018). It is intuitive to imagine that after a species geographically expands, increases in speciation rates could easily result from the synchronous creation of physical barriers, such as the rise of mountain chains or expansion and retraction of habitats driven by changes in temperature (e.g., Lagomarsino et al., 2016; Rangel et al., 2018; Vrba, 1993). For this reason, we decided to focus our discussion on geographical opportunities mediated by environmental heterogeneity because the role of geographical barriers is better understood (see also Abe & Lieberman, 2009; Brown et al., 2013; Kozak et al., 2006). Assuming that a species is able to expand geographically, we argue that geographic opportunities mediated by environmental heterogeneity might generate a burst in speciation rates even without a pulse in physical barriers, but for that to occur certain population‐level and microevolutionary conditions/scenarios are necessary. This pulse in speciation and, thus, an increase in speciation rates, might be produced by parapatric speciation driven by environmental heterogeneity if the time for geographic expansion of a species (*t*_exp_) is shorter relative to the time its populations take to evolve reproductive isolation (TTBS) (*t*_exp_ < TTBS, Φ < 1; Figure 2a,b). In other words, if a species has “enough time” to expand geographically and reach several geographical opportunities before reproductive isolation emerges, it might allow for a synchrony in several speciation events producing an increase in speciation rates observed at the macroevolutionary scale. How fast a lineage can expand geographically has been indeed suggested by several authors as a potential limiting step in speciation (Herrera‐Alsina et al., 2018; O'Donovan et al., 2018; Price et al., 2014; Weir & Price, 2011). However, even if a species expands more rapidly than the time it takes for reproductive isolation to evolve (Φ < 1), diversification patterns might still differ depending on how fast is the emergence of reproductive isolation (e.g., left and right lower corners in Figure 2a,b).

On one hand, when a species can rapidly expand geographically and is also able to readily produce new species (very small *t*_exp_ and TTBS; lower left corner in Figure 2a,b), we might identify this as a rapid diversification. If carrying capacity is present, this rapid diversification would attain an “early burst” pattern (lower left corner in Figure 2a). If this limit is absent, opportunities to speciate will not be limited and the radiation will keep constantly generating species (lower left corner in Figure 2b). This rapid diversification scenario, where both the time of expansion and the time that reproductive isolation takes to evolve are short, might represent the process underlying population differentiation along environmental gradients in different taxa (e.g., Friis et al., 2018; Hecht et al., 2015; Walter et al., 2020). The Oregon Junco, for example, has diverged less than 15.000 years ago, already has a distribution encompassing a wide latitudinal range (from Baja California to Alaska), and also shows high population differentiation across space (Friis et al., 2018) representing a good candidate for the above scenario. Plants of the genus *Senecio* comprise another important example (see a review in Walter et al., 2020). The *S. lautus* complex occurs in very distinct habitats and has locally adapted, and mechanisms such as natural selection against hybrids are likely leading to rapid speciation (Walter et al., 2020).

On the other hand, if a given species rapidly expands through the geographical area but takes considerably longer to generate new species compared with the above scenario (TTBS >>>> *t*_exp_, Φ <<<< 1; right lower corner in Figure 2a,b), we would still be able to see an increase in speciation rates. However, in this case, the radiation would show a distinct pattern. Here, speciation events would take place later in the radiation, characterizing what we here called a “late burst” in speciation rates. This late burst pattern could happen either in a scenario with or without carrying capacity (Figure 2a,b). This “late burst” is expected because all the new species originate from the same “mother” lineage at similar points in time, leading to a phylogeny with a short waiting time interval between each splitting event (short BSI, Figure 1b) (see also Moyle et al., 2009 for a possible case). Thus, although reproductive isolation takes relatively longer to evolve, the resulting diversification process would still present very short waiting times between speciation events suggesting a burst in speciation at the macroevolutionary scale (Figure 1b, Figure 2a,b). One could view this scenario as one where multiple populations start “ticking” their “speciation clock” at around the same time but the clock is rather slow (Figure 1b). Empirical examples could comprise species that have recently spread across an area, already comprise several distinct populations, and reproductive isolation is slowly and synchronically evolving among populations. We argue that this “late burst” scenario might help to understand why rates measured at the microscale and macroscale are not necessarily expected to be coupled (see Box 2).

**When micro does not meet macro: the relationship between rates of reproductive isolation and speciation**

Speciation rates measured at the macroevolutionary scale vary enormously across lineages and partially explain why some groups of organisms are more species‐rich than others. What are the population‐level processes controlling this variation in biological diversity have been increasingly debated (e.g., Harvey et al., 2017, 2019; Rabosky, 2015; Rabosky & Matute, 2013; Singhal et al., 2018) and are the focus of this piece. Evolutionary biologists frequently consider reproductive isolation as the limiting step of the speciation process (see Coyne & Orr, 2004; Nosil, 2012) and, thus, it is feasible to expect that reproductive isolation would also control the speciation rates that we measure at the macroscale. Therefore, all else being equal, clades with higher rates of reproductive isolation should also comprise higher speciation rates.

Rabosky and Matute (2013) were the pioneers in empirically testing this prediction. These authors compared the relationship between speciation rates and rates of reproductive isolation in clades of birds (postzygotic) and flies (pre and postzygotic). Surprisingly, they found that although reproductive isolation evolves faster in some species than in others, this variation was not related to the speciation rates (Figure I). The authors conclude by highlighting that the persistence of incipient species (see Box 1) might be an important mechanism limiting speciation rates (as suggested by Rosenblum et al., 2012 and Dynesius & Jansson, 2013, and reviewed in Harvey et al., 2019).

Our “late burst” scenario (see text for details, Figures 1 and 2a,b) could also partially explain this lack of relationship. In this “late burst” scenario, the short BSI, and hence, very high speciation rates measured at the macroevolutionary scale contrast with the microevolutionary temporal pattern of a long time for species to be formed (they all had a long and similar TTBS) (see Figure 1b). If an analysis such as the one done by Rabosky and Matute (2013) simultaneously includes clades that have undergone diversification dynamics under the “late burst” scenario and under a gradual process (compare Figure 1a,b), we expect that no positive relationship will emerge between micro and macro rates: a clade diversifying under the gradual process (Figure 1a) has a comparatively higher rate of reproductive isolation (and hence shorter TTBS) and a lower speciation rate (and hence longer BSI); A clade diversifying under the “late burst” scenario (Figure 1b) has a comparatively lower rate of reproductive isolation (and hence longer TTBS) but a higher speciation rate measured at the macroevolutionary scale (and hence shorter BSI).

We note that the comparisons and discussions made assume a “species phylogeny”, but even if we empirically access something more akin to the “gene phylogeny”, the argument comparing microevolutionary and macroevolutionary rates remains the same, although an early burst is produced in the reconstructed gene phylogeny (Figure 1b). Finally, it is important to note that the “late burst” scenario was partially mentioned by Coyne and Orr (2004) but not further discussed as a relevant and/or common process, especially in the context of exploring how increases in speciation rates could be generated and how this scale up to broad macroevolutionary patterns.

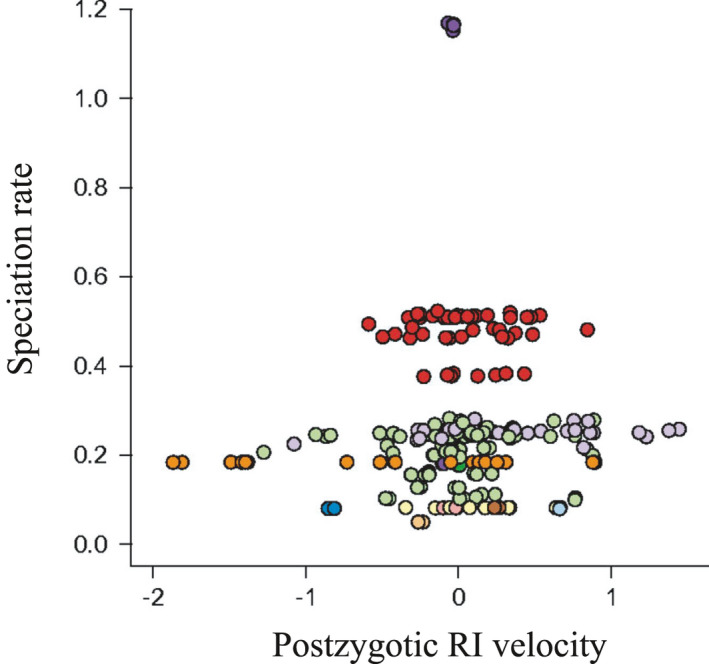

**FIGURE I** Species of birds that evolve intrinsic reproductive isolation faster do not have higher rates of speciation. Each point is a different species and the distinct colors correspond to distinct bird orders. RI: reproductive isolation. Reproduced from Rabosky and Matute (2013), with permission

Finally, when it takes longer for a species to conquer geographical space than to evolve reproductive isolation (TTBS < *t*_exp_, Φ > 1), we would expect to see gradual (as opposed to clustered) speciation events, paced by the time a given clade spreads in space (see cartoon phylogenies within the panels above the 1:1 line, Figure 2a,b). In other words, as populations of a species gradually colonize distinct environments and reach distinct adaptive peaks, reproductive isolation emerges, and neither a synchrony in speciation events (a pulse) or increases in speciation rates would be produced. This might happen, for example, if incumbent species (Rosenzweig & McCord, 1991; Schenk et al., 2013; Van Valkenburgh, 1999) and/or insufficient genetic variation in populations (Polechová, 2018; Polechová & Barton, 2015) prevent the rapid spread of a species across space. Under this hypothetical scenario, bursts in speciation within the lineage of interest are unlikely to be produced, either in the presence or absence of carrying capacity (Figure 2a,b). Important to note that reproductive isolation can indeed evolve fast in some organisms (e.g., Coyne & Orr, 1989; Saulsberry et al., 2017), and that even if range expansion is fast, it might be relatively slower than TTBS, generating a Φ larger than one.

### 3.2 The tempo and mode of speciation dynamics driven by resource‐partitioning opportunities

When speciation events are solely mediated by resource‐partitioning opportunities (Figure 2c,d) (Box 1), the time that a species takes to geographically expand (*t*_exp_) does not affect speciation rates. Independently of being able to quickly cover a geographical area (small or higher values of *t*_exp_), populations of a given species will not face any geographic opportunity. This might happen, for example, in small isolated islands where a species can rapidly expand geographically but no (or very few) geographic opportunities are present. Scenarios in Figure 2c,d, more generally, represent a radiation where competition leads to the adaptive differentiation in resource utilization and speciation happens in sympatry (Martin & Richards, 2019; Stroud & Losos, 2016; Yoder et al., 2010). This mechanism is relevant to partially explain the diversification dynamics of macroevolutionary patterns underlying several adaptive radiations, especially those in islands and lakes, such as Darwin's Finches, Caribbean Anoles, or Cichlids (Grant & Grant, 2008; Losos, 2009; Seehausen, 2015).

In the absence of geographical opportunities, reproductive isolation would have to rapidly evolve within populations for increases in speciation rates to be produced (left side of Figure 2c,d). This is because populations are undergoing resource‐partitioning as a result of competition and need to rapidly differentiate to coexist. When TTBS is high, populations would take such a long time to differentiate that they might become extinct by competitive exclusion before becoming distinct species, or they might merge back together. As we can observe in Figure 2c,d, when resource partitioning is the rule, bursts in speciation rates might be easier to be produced when TTBS is short (Figure 2c,d), a more restrictive condition compared with the geographical opportunities scenario (Figure 2a,b). The presence of ancient adaptive alleles (the transporter hypothesis), among other population‐level mechanisms, might have an important role in explaining how resource‐partitioning could rapidly generate population differentiation, reproductive isolation and increases in speciation rates (Marques et al., 2019; Martin & Richards, 2019).

Similar to the geographical opportunities scenario, different patterns of speciation rates would be detected depending on whether or not a species carrying capacity is present (Figure 2c,d). When a cap in diversity is present, speciation events will be clustered early in the radiation of a clade—suggesting an “early burst” pattern measured at the macroevolutionary scale—because the lineage rapidly diversifies, reaches its carrying capacity and speciation opportunities slowdown as time goes by (Figure 2c). On the other hand, when there is no limit on the number of species, we expect to observe several speciation events taking place but no sign of rate slowdown (Figure 2d).

3.3

As we see, the theoretical scenarios we describe above (geographical and resource‐partitioning opportunities) might produce different macroevolutionary patterns such as gradual speciation events, early bursts (or diversification slowdown), and late bursts. Although we frequently mention that bursts in speciation rates reflect the synchrony of the time that reproductive isolation takes to evolve among populations (TTBS), the emergence of reproductive isolation is not expected to be perfectly synchronous among these populations. Populations will, of course, slightly differ in their “speciation clock” but these temporal differences will be minimal when observed at the macroevolutionary scale.

It is also important to acknowledge that the macroevolutionary patterns depicted in Figure 2 are not an exclusive outcome of our proposed scenario and do not necessarily directly match expectations of what we should detect in molecular phylogenies. The protracted speciation model can predict diversification slowdowns as measured by molecular phylogenies (Rosindell et al., 2010; Etienne & Rosindell, 2012; Etienne et al., 2014; see also Box 1). This model is based on the idea that speciation is not an instantaneous event, focusing on the time speciation takes to be completed (TTBS in our framework) and how that affects the signal of a slowdown measured in molecular phylogenies. Usually, such slowdown is positioned very close to the present, which suggests that they might be quite different from a scenario where the slowdown has left a signal through most of a given clade's history. Our framework, on the other hand, discusses how population‐level, in particular geographical expansion, and microevolutionary aspects interact to generate a synchrony in speciation events and predict diversification slowdowns in the “real” diversification of lineages. Hence, our framework builds upon the protracted speciation idea and further explores the underlying mechanisms behind the idea that speciation is not an instantaneous event. Given enough time, our model also predicts a slowdown through most of the clade's history and not only closer to the present. This prediction can be tested by “chopping” out the very recent lineages, although it might not be an easy task to decide how far back we should go in an empirical phylogeny.

## 4 THE GEOGRAPHICAL SETTING AND INTRINSIC PROPERTIES OF THE ORGANISMS MODULATE BOTH POPULATION‐LEVEL AND MICROEVOLUTIONARY PROCESSES

Up to now, our discussion has focused on general population and microevolutionary properties driving macroevolutionary patterns as a tool to explore general principles. However, organisms occur across a wide diversity of landscapes around the globe, have completely different morphologies and ecologies, and exhibit a huge variation in life‐history aspects. Moreover, researchers are interested in comparing diversification dynamics of different organisms (e.g., Román‐Palacios & Wiens, 2018) or to compare the diversification of the same lineage at different geographical settings (e.g., Pinto et al., 2008). Hence, understanding how those different organismal and environmental aspects produce or modulate the different underlying population‐level and microevolutionary processes will help us to further understand the tempo and mode of speciation. Here, we discuss how two interconnected factors—habitable area and dispersal abilities—might affect demographic mechanisms and microevolutionary processes and consequently change the course of a radiation.

Among the most obvious properties of a given geographical setting is its total area. A larger available area might allow species to attain a larger geographic range, increasing the probability of vicariant events and the chance of populations to experience very different environments (Kisel et al., 2011; Losos & Parent, 2009; Moen & Morlon, 2014; Rosenzweig, 1995). Hence, larger areas might harbor more geographic opportunities and should foster in situ speciation, increasing the likelihood of several speciation events to take place in a short interval of time (Kisel et al., 2011; Losos & Parent, 2009; Losos & Schluter, 2000; Schluter & Pennel, 2017), the scenarios depicted in Figure 2a,b. This perspective might be evident when trying to compare continental and insular radiations for the same lineage.

It is important to say, however, that “large and small” (concerning the area in this case) are not universal qualities but strongly dependent on how different organisms perceive the world. Take the bat, *Eptesicus fuscus*, and the white‐footed mice, *Peromyscus leucopus*, studied by Richardson et al. (2020), for example. The bat has a much higher dispersal ability compared with the mice, resulting in disparate genetic patterns between the two species, despite living in the same area (Richardson et al., 2020). If we were able to follow the range expansion of these two species since their first arrival in the area, *t*_exp_ would likely differ between these two species. This is because *t*_exp_ is the ratio of “area” and “expansion rate”, the latter likely to be strongly influenced by organismal properties such as dispersal abilities. Therefore, a large geographical area might seem “small” for a given species if it has a very high dispersal ability and is able to quickly reach the whole area (e.g., “the bat”). Under this scenario, the mechanisms we proposed here predict a burst in speciation when geographical opportunities are mediated by environmental heterogeneity and there is strong local selection to counteract gene flow (e.g., Walter et al., 2020). On the other hand, if the species' dispersal abilities are lower (e.g., “the mice”), more time would be necessary for all geographical opportunities to be fully exploited. In this last case, a burst in speciation rates could still be generated if the time that populations take to evolve reproductive isolation is considerably higher relative to the long time taken for geographic expansion to occur (making Φ < 1, Figure 2a,b). Another route for “the mice” to produce a burst in speciation rates would be if resource‐partitioning opportunities are available and, in this case, the time for geographic expansion would not matter (Figure 2c,d).

The perception of the interplay between area and dispersal abilities, as well as an understanding of the underlying mechanism driving speciation opportunities, might also help to shed some light in the discussion of whether speciation is promoted/limited by higher dispersal abilities (Claramunt et al., 2012; Czekanski‐Moir & Rundell, 2019; Kisel & Barraclough, 2010; Kisel et al., 2011; Weeks & Claramunt, 2014). While higher dispersal abilities might allow populations of a given lineage to reach the opportunities to speciate across space by shortening *t*_exp,_, it could also prevent population differentiation and speciation because of recurrent gene flow (Claramunt et al., 2012; Kisel & Barraclough, 2010). Similarly, higher dispersal abilities can also increase the potential for partially reproductively isolated species to come into secondary sympatry, which could either accelerate the speciation process by reinforcement and character displacement, or prevent speciation to be completed (see McEntee et al., 2018; Weir & Price, 2011). Although intermediate dispersal abilities have been suggested to increase speciation rates by allowing organisms to expand their distributions but not genetically homogenize their populations (see Czekanski‐Moir & Rundell, 2019; Weeks & Claramunt, 2014), the extent to which dispersal ability might prevent or facilitate speciation remains an open empirical question, and its effect might vary with the spatial scale, across different groups, and depend upon the strength of divergent selection acting (Claramunt et al., 2012; Gavrilets & Vose, 2005; Kisel & Barraclough, 2010; Kisel et al., 2011).

However, when we think about radiations taking place within a single landmass area (e.g., a continent), it is possible that studies that eventually show a negative association between dispersal abilities and speciation rates (e.g., Claramunt et al., 2012) will more likely include lineages that preferentially speciate by geographical opportunities produced by physical barriers. The reasoning rests on the idea that if speciation is mostly driven by geographical barriers, lineages with very high dispersal abilities might eventually homogenize populations after these barriers emerge, preventing speciation. On the other hand, studies that eventually find a positive association between dispersal abilities and speciation (potentially Phillimore et al., 2006) will more likely include lineages that typically speciate by geographical opportunities mediated by environmental heterogeneity. Here, local selection would be strong enough to overcome the potential genetic homogenization caused by the higher dispersal abilities of organisms, allowing the speciation process to be completed. One could view these arguments as similar to those reviewed by Weeks and Claramunt (2014), where different modes of speciation (isolation‐limited models vs. founder‐event models) could explain the dual effect that has been suggested for dispersal abilities on speciation. However, there is an important difference. In the arguments based on the isolation‐limited and founder‐event models, speciation is limited by the efficacy of geographical barriers to keep populations isolated versus to speciation being limited by the ability of lineages to geographically expand, respectively. On the other hand, our arguments for the dual effect of dispersal ability on speciation rates rely on speciation being either mediated by physical barriers or by environmental heterogeneity, after the lineage has geographically expanded in both cases. Hence, our framework reinforces that a fruitful venue to understand the effect of dispersal abilities on speciation rates might be to investigate the nature of the speciation processes at the local scale. This could allow one to disentangle reproductive isolation from being driven by the interplay between strong local selection and dispersal abilities, versus reproductive isolation emerging as a product of the nature of the physical barriers and species dispersal abilities, accompanied or not by natural selection. Therefore, our framework also emphasizes the potential role that natural selection might have on the relationship between dispersal abilities and speciation rates.

Examples illustrated above reinforce the idea that it is the interaction between the geographical settings and the intrinsic properties of the organisms that modulate population and microevolutionary processes, ultimately determining the macroevolutionary rates. In this respect, rates of speciation (and extinction) should be seen as emergent properties that arise from the interplay between intrinsic and extrinsic factors. This conceptualization of rates as emergent properties has been previously discussed (Jablonski, 2008a, 2008b; Rabosky, 2013) and might even be a common sense, but there are some practical implications that we think might not have been fully appreciated. For example, we suggest that macroevolutionary comparative studies should not only take into account phylogenetic relationships (to “control” for intrinsic properties of the organisms) but also take into account environmental similarity. Many macroevolutionary studies, for example, try to find the “trait‐related” or “environmental‐related” speciation rate (e.g., all the xSSE models, Fitzjohn, 2010), either by looking for the effect of “species traits” (MuSSE or akin models) or the “environmental effect” (e.g., GeoSSE and akin models, Goldberg et al., 2011). Although some studies indeed explore the importance of both factors in driving the radiation of a single lineage (e.g., Condamine et al., 2018; Lagomarsino et al., 2016; Wagner et al., 2012), their interaction is still not fully integrated into the statistical structure of the analysis in typical macroevolutionary studies. These different statistical concerns (phylogenetic effects vs. spatial autocorrelation effects) map into two broad areas of expertise. While macroevolutionary studies typically correct for phylogenetic structure (Felsenstein, 1985; Harvey & Pagel, 1991), macroecological studies correct for spatial autocorrelation (e.g., Legendre, 1993). If we aim to see macroevolutionary rates as emergent properties of intrinsic and external factors, we should also include in our macroevolutionary analytical toolkit the spatial autocorrelation aspect of rates when trying to study what explains rate heterogeneity.

## 5 FUTURE DIRECTIONS

A fruitful next step would be to build a dynamic model that incorporates the parameters from our index. This would allow researchers to investigate whether the interplay between the time a species takes to geographically expand and the time that reproductive isolation takes to evolve indeed result in the macroevolutionary patterns we predict (Figure 2). Individual‐based and spatially explicit models have indeed provided some valuable insights into how microevolutionary processes might shape the macroevolutionary patterns produced by rapidly multiplying lineages (e.g., Aguilée et al., 2018; Costa et al., 2019; Gavrilets & Vose, 2005; Rangel et al., 2018). These studies have actually provided some theoretical support for several of the points raised here, such as the potential role of environmental heterogeneity in driving speciation bursts, the importance of distinct levels of gene flow in modulating macroevolutionary patterns, and the importance of the geographical context and competitive interactions in driving the diversification of clades. Studies on how the interplay of population‐level and microevolutionary mechanisms might lead to macroevolutionary patterns are still in their infancy but those will certainly help us to properly build the bridge between microevolution and macroevolution.

The ideas presented here should be viewed as a starting point to motivate more realistic models. For example, when building a model based on the ideas proposed here, parameters would not have to be fixed and both *t*_exp_ and TTBS could attain different values during the radiation of a lineage. For the sake of our arguments, we assumed that TTBS is constant within a given clade of interest, either among its lineages or in time, but as far as the relationship between TTBS and BSI does not drastically change (e.g., TTBS > BSI to TTBS < BSI) in different portions of the tree, our rationale should be fine. If TTBS varies considerably either among lineages or in time, enough to change the relationship between TTBS and BSI in different portions of the tree, then a more complex scenario emerges. Future work could incorporate this aspect.

A dynamic framework would also allow one to take into account potential shifts in the total habitable area, which is likely to occur given changes in climate and in the environment (e.g., Jetz & Fine, 2012; Rangel et al., 2018). The interplay between resource‐partitioning and geographical opportunities availability could also be modeled. Although those are here treated in two opposite scenarios, it is expected that both are present during the diversification of a lineage (e.g., Aguilée et al., 2018; Lagomarsino et al., 2016; Vrba, 1987). Going further, this dynamic model could also incorporate the effects of intraspecific competition, which might influence the spread of populations across space (e.g., Grabowska et al., 2019). Adding extinction at population and species levels would also be an important step given that extinction dynamics is certainly an extremely important aspect at macroevolutionary scales (Dynesius & Jansson, 2013; Jablonski, 2005; Rabosky, 2013; Raup, 1994). Going further, although we have here focused on exploring the interaction of species geographic expansion and speciation, a separate model focusing on resource partitioning could exchange “*t*_exp_” for “t_morpho_” to directly investigate the time it takes for the morpho/ecospace to be fully occupied. A model simultaneously having both axes (geographical and morphological) should also be further explored as those are likely to affect each other.

However, going beyond models and simulations and exploring our framework in real biological systems will be challenging. Specifically, empirically measuring the elements of the numerator will not be straightforward. One possible avenue could be to use niche modeling approaches (see Kozak et al., 2008) to estimate the “habitable area” of a group, how this area has changed over time, and the proportion of the area conquered since the onset of the group diversification. Alternatively, one could view a lineage's current geographical distribution as the total area in an exercise to try to retrospectively infer the other parameters discussed here. After all, this is the total area occupied that presumably produced the extant diversity of the lineage. The temporal reconstruction of the environment might also shed some light on how one could model changes in the area and help to overcome the assumption of having a fixed total area (e.g., Jetz & Fine, 2012). Proxies for species dispersal abilities (e.g., hand‐wing index for birds, see Weeks & Claramunt, 2014) could also be used as a crude proxy for the rate of geographic expansion. However, caution should be taken given that these proxies only take into account the species properties and not the external environment when “measuring” the potential for geographical expansion. Furthermore, TTBS or related proxies are available for a few lineages and taxonomic groups (e.g., some birds and flies, Rabosky & Matute, 2013), which could be either intrinsic postcopulatory incompatibilities, precopulatory mechanisms or even related to hybrid maladaptations. However, to be able to fully empirically explore our proposed framework we still need data on the timing of reproductive isolation and genetic differentiation for a much larger number of lineages.

## 6 CONCLUSIONS

Here, we have discussed how population‐level and microevolutionary processes might interact to produce macroevolutionary patterns to better understand and predict when gradual or bursts in speciation dynamics might be produced. In summary, the four main points raised by our perspective are:

1. A pulse in speciation might be produced by parapatric speciation driven by environmental heterogeneity if the time that a species takes to geographically expand is shorter relative to the time its populations take to evolve reproductive isolation.
2. An increase in speciation rates produced by geographical opportunities mediated by environmental heterogeneity is more likely to occur in larger areas where higher environmental heterogeneity is expected to occur. However, one should note that the intrinsic properties of the organisms should modulate how an organism perceives the environment.
3. Therefore, bursts in speciation rates (or any rate for that matter) represent an emergent property that arises from the interaction between lineages and environmental properties. Although this conceptualization is not necessarily new, there are some practical implications that have not been fully appreciated in macroevolution. In particular, this suggests that when trying to compare the diversification dynamics of different lineages, a comparative analysis should not only control for phylogenetic relatedness but also for spatial autocorrelation of a clade's environment.
4. When bursts in speciation rates result from geographical opportunities mediated by environmental heterogeneity, and the time populations take to evolve reproductive isolation is longer than the time the species takes to expand geographically, rates of reproductive isolation measured at the microevolutionary scale might not predict speciation rates measured at the macroevolutionary scale.

The examples discussed here are far from attaining all possibilities, but we hope to have drawn attention to interesting mechanisms to be further studied and to motivate the development of new theoretical models, in particular those aiming to investigate the interplay between the time a lineage takes to geographically expand and the time it takes to evolve reproductive isolation. However, to empirically test the ideas proposed here we still have a long way to go on generating a huge amount of data for a wide range of groups, not only regarding their genetic aspects but also regarding their demographic properties and their often poorly known natural history.

## CONFLICT OF INTEREST

The authors declare no competing interests.

## AUTHOR CONTRIBUTIONS

**Laura Rodrigues Vieira de Alencar:** Conceptualization (equal); funding acquisition (equal); project administration (equal); visualization (equal); writing‐original draft (equal); writing‐review & editing (equal). **Tiago Bosisio Quental:** Conceptualization (equal); funding acquisition (equal); project administration (equal); visualization (equal); writing‐original draft (equal); writing‐review & editing (equal).

## Data Availability

There are no data to be archived.
